# Advanced Maternal Age, Mode of Delivery, and Thyroid Hormone Levels in Chinese Newborns

**DOI:** 10.3389/fendo.2019.00913

**Published:** 2020-01-10

**Authors:** Pianpian Fan, Zhong-Cheng Luo, Ning Tang, Weiye Wang, Zhiwei Liu, Jun Zhang, Fengxiu Ouyang

**Affiliations:** ^1^Ministry of Education and Shanghai Key Laboratory of Children's Environmental Health, Xinhua Hospital, Shanghai Jiao Tong University School of Medicine, Shanghai, China; ^2^Department of Obstetrics and Gynecology, Prosserman Centre for Population Health Research, Lunenfeld-Tanenbaum Research Institute, Mount Sinai Hospital, Institute of Health Policy, Management and Evaluation, University of Toronto, Toronto, ON, Canada; ^3^Department of Neonatology, International Peace Maternity and Child Health Hospital, Shanghai Jiao Tong University School of Medicine, Shanghai, China

**Keywords:** maternal age, mode of delivery, perinatal factors, thyroid hormones, Chinese newborns, birth cohort

## Abstract

**Objective:** Thyroid hormones are essential for fetal growth and neurodevelopment, however, data on cord blood thyroid hormones are sparse in China where maternal age at childbearing is increasing in recent decades. We aimed to assess cord blood levels of free triiodothyronine (FT3), free thyroxine (FT4), and thyroid stimulating hormone (TSH) in full-term Chinese newborns, and examine potential related perinatal factors.

**Methods:** This study included 922 mother-newborn pairs from a prospective birth cohort enrolled in 2012–2013, Shanghai, China. Cord serum concentrations of FT3, FT4, TSH, and TPOAb were measured in newborns.

**Results:** Newborns born via cesarean section had higher cord serum FT3 (mean ± SD: 1.90 ± 1.16 pmol/L) and lower cord serum TSH (5.15 ± 2.60 mIU/L) than those born via vaginal delivery (FT3: 1.62 ± 0.93 pmol/L; TSH: 9.27 ± 6.76 mIU/L). In cesarean section deliveries, the concentration of cord serum FT3 was 0.15 (95%CI: −0.03, 0.33; *p* = 0.10) pmol/L lower in infants of mothers aged 30–34 years, and 0.57 (95%CI: 0.22, 0.92; *p* = 0.002) pmol/L lower in infants of mothers ≥35 years compared to infants of mothers <30 years. Large-for-gestational-age (birth weight >90th percentile) was associated with higher TSH (*p* = 0.02). Similar results were also found in vaginal deliveries.

**Conclusions:** In this Chinese term birth cohort, newborns born via cesarean section had higher cord serum FT3 and lower TSH than those born via vaginal delivery. Advanced maternal age was associated with lower fetal FT3. Further research is needed to understand whether this association may mediate the adverse impact of advanced maternal age on neurodevelopment in early life.

## Introduction

Thyroid hormones, thyroxine (T4), and triiodothyronine (T3) are essential for fetal growth and neurodevelopment (1). From mid-gestation, fetal hypothalamus-pituitary-thyroid axis becomes gradually functional, and produces increasingly more fetal thyroid hormones including free triiodothyronine (FT3) and free thyroxine (FT4) (2). Thyroid peroxidase antibody (TPOAb) is thyroid autoantibody which is not functional antibody and merely reflects autoimmunity (3). Neonatal TPOAb are mainly from maternal origin (4). The positive TPOAb in cord blood has been associated with a higher risk of developing autoimmune thyroiditis in children and adolescents (5). In the context of newborn screening programs for congenital hypothyroidism (CH), thyroid stimulating hormone (TSH) (6, 7), or T4 levels (8, 9) or both have been used (10), while FT3, FT4, and TPOAb levels are not routinely measured.

Neonatal thyroid function screening measures TSH levels in either cord blood or heel prick blood at 48–72 h after births (11). The heel prick approach is somewhat compromised by the lack of consistent procedures in clinical practices, and TSH level may also be affected by the timing of blood collection and temperature (6, 12). Cord blood sampling is convenient and non-invasive. Some studies have provided the reference values for thyroid hormones in cord blood (13–16). Data on thyroid hormones in cord blood are sparse in China.

Cord blood TSH levels are elevated among neonates who have endured intrapartum stress including the induction of labor (17), long duration of labor (7), and vaginal delivery (7). However, inconsistent associations with neonatal thyroid hormones have been observed for maternal factors including gestational hypertension, preeclampsia, and gestational diabetes mellitus (GDM) (8, 18–20), and birth outcomes (15, 21–24). Inadequate adjustments for delivery factors may partly explain previous inconsistent results (20).

Advanced maternal age, commonly defined as ≥35 years at the time of childbirth (25, 26), has become increasingly common over recent decades in China and many other countries, but its impacts on neonatal/cord blood thyroid hormone levels are not well-characterized. A study found that maternal age >30 years was associated with lower maternal serum FT3 and FT4 levels in the first and second trimesters of pregnancy (27). The rates of cesarean section (C-section) are high in China (68.7% in Shanghai in the year 2009), in part due to frequent C-section on request (without medical indications) (48.4%) (28). In the World Health Organization Global Survey between 2004 and 2008, C-section rate in China was 46.2%, the highest among 24 study countries (29). This provides a unique opportunity to explore advanced maternal age and other perinatal factors in relation to fetal thyroid hormone levels in physiological conditions.

In this study, we sought to describe cord blood FT3, FT4, TSH, and TPOAb levels measured via chemiluminescent microparticle immunoassay in full-term neonates of Chinese mothers with normal thyroid function from a Shanghai birth cohort, and evaluate the impacts of maternal age, mode of delivery and other perinatal factors.

## Methods

### Study Design and Participants

The data of this study was from the Shanghai Obesity and Allergy Cohort, a prospective birth cohort recruiting participants in two tertiary care hospitals in Shanghai, China, 2012–2013 (30). The cohort study is designed to evaluate the effects of environmental exposures and mode of delivery on childhood obesity and allergic diseases. Women with a singleton pregnancy at gestational age ≥28 weeks were recruited. The study was approved by the Medical Ethics Committee of Xinhua Hospital, Shanghai Jiao Tong University School of Medicine. Written informed consent was obtained from all study participants.

Cord blood serum FT3, FT4, TSH, and TPOAb were measured. We excluded infants of mothers with syphilis (*n* = 7), artificial fertilization (*n* = 28), preterm deliveries (*n* = 33), and infants of mothers without information on thyroid function (*n* = 22). Also, infants of mothers with thyroid diseases (hyperthyroidism, *n* = 10; hypothyroidism, *n* = 26) were excluded (31). The thyroid diseases were based on clinical diagnosis in medical charts. Thus, the final study sample was 922 singleton newborns.

### Measurements of Cord Serum FT3, FT4, TSH, and TPOAb

Immediately after delivery, cord blood sample was collected and put into serum separation tubes. The tubes were rested for 30 min at room temperature before centrifugation to allow coagulation. Aliquotes of serum samples were stored at −80°C until assays.

Cord blood serum FT3, FT4, TSH, and TPOAb concentration were measured by chemiluminescent microparticle immunoassay using the Architect system (Abbott Laboratories, Abbott Park, IL, USA) in the clinical laboratory of the Shanghai International Peace Maternity and Child Health Hospital of Chinese Welfare Foundation; the lab is certified by the China National Accreditation Board. QA/QC procedures were performed for all analyses in accordance with the system's instructions. The inter- and intra assay coefficients of variation were 5.9 and 5.0–5.1% for FT3, 2.5–6.3 and 3.5% for FT4, 2.5–4.1 and 2.2–2.9% for TSH, respectively. The limits of detection (LOD) for FT3, FT4, TSH, and TPOAb were 1.54 pmol/L, 5.15 pmol/L, 0.01 mIU/L and 0.5 IU/mL, respectively. The cross-reactivity was 0.002% between T3 and T4 assays for FT4 > 1000,000 pg/mL, and 0.0035% for FT3 > 12,000 ng/dL. Therefore, the assays showed virtually no cross-reactivity between FT3 and FT4 in the present study. The detectable TPOAb was ≥0.5 IU/mL. The TPOAb positive was defined as ≥ 5.61 IU/mL.

### Maternal and Delivery Factors

A face-to-face questionnaire interview was conducted at enrollment to collect data on maternal characteristics including age, weight before pregnancy, height, education, annual household income, parity, and smoking and passive smoking (husband smoking) during pregnancy (there were only 2 women who smoked in the cohort). Pregnancy complications and comorbidities, the use of artificial fertilization, mode of delivery [vaginal delivery, C-section (medical-indicated or on-request /non-medical-indicated)], type of labor (spontaneous or induced), duration of labor (the first and second stages) were abstracted from medical charts using a standardized data collection form.

The diagnoses of hyperthyroidism and hypothyroidism were made by the treating obstetrician based on the Chinese guidelines for the diagnosis and treatment of thyroid diseases in pregnancy (31). Preeclampsia was diagnosed by obstetricians (32). GDM was defined according to the International Association of Diabetes and Pregnancy Study Groups (IADPSG) criteria (33).

C-section deliveries were divided into two categories, medical-indicated and non-medical-indicated. Non-medical-indicated C-section was defined as a C-section on maternal request in the absence of medical indications, and medical-indicated C-section was defined as a C-section on maternal and/or fetal indications (34). The duration of first stage of labor was defined as the duration between the onset of regular painful uterus contractions and full dilatation of the cervix. The duration of second stage of labor was defined from complete cervical dilation to delivery of the neonate (35). Long duration of labor during the first or second stage was defined as >75th percentile.

Advanced maternal age was defined as age ≥35 years (25). Maternal age was categorized into 3 groups: <30, 30–34 and ≥35 years. Pre-pregnancy body mass index (BMI) was calculated as pre-pregnancy weight divided by the square of height (kg/m^2^), and categorized according to the World Health Organization (WHO) criteria: underweight, <18.5 kg/m^2^; normal weight, 18.5–24.9 kg/m^2^; overweight and obesity, ≥25.0 kg/m^2^ (36). Maternal education levels were grouped into two categories: high school or lower; college/university.

### Neonatal Factors

Infant sex, 5-min Apgar score, gestational age (GA) at delivery and birth weight were obtained from medical charts.

GA at delivery was determined by the date of last menstrual period (LMP) or early fetal ultrasound dating (<20 weeks). When the difference in GA estimates by the two methods was ≥2 weeks, the ultrasound-based estimate was used. Birth weight was categorized as low (LBW, <2,500 g), normal (2,500–4,000 g) and high (HBW, >4,000 g). Birth weight for GA was defined according to the Chinese references for male and female newborns separately (37), and classified as small for GA (SGA, <10th percentile), appropriate for GA (AGA, 10th−90th percentile), and large for GA (LGA, >90th percentile).

### Statistical Analyses

Descriptive statistics (mean, SE, median, and selected percentiles) were presented for cord blood FT3, FT4, TSH, TPOAb, and FT3/FT4 ratio. We calculated FT3/FT4 ratio as FT3 divided by FT4, which is a proxy of deiodinase activity (38). The outcome variable was each of the cord blood thyroid parameters. Logistic regression was used for TPOAb detectability as the outcome variable, and linear regressions were used for cord blood FT3, FT4, TSH, and FT3/FT4 ratio as the outcome variables. To examine the association with maternal age, the models were adjusted for pre-pregnancy BMI (<18.5, 18.5–24.9, ≥25.0 kg/m^2^), hypertensive disorders of pregnancy (chronic hypertension, gestational hypertension, preeclampsia, and none), GDM or pre-existed diabetes, infant sex and gestational age. To examine the associations with mode of delivery, induction of labor and duration of first or second stage of labor (among vaginal deliveries only), the models were additionally adjusted for maternal age (<30, 30–34, and ≥35 years), and parity. To examine the association with fetal growth, the models were adjusted for maternal age (<30, 30–34, and ≥35 years), pre-pregnancy BMI (<18.5, 18.5–24.9, ≥25.0 kg/m^2^), hypertensive disorders of pregnancy, GDM or pre-existed diabetes, infant sex, and gestational age.

The primary association of interest is whether advanced maternal age is associated with cord blood thyroid parameters (FT3, FT4, TSH levels, FT3/FT4 ratio, and TPOAb). Two-sided *p* < 0.025 were considered statistically significant. All analyses were performed using the SAS 9.2 software (SAS Institute, Inc, Cary, NC, USA).

## Results

### Study Population

This study included 922 mothers without thyroid diseases and their newborns. The characteristics of the study cohort are shown in Table 1. There were 97.3% mothers of Han Chinese, 5.6% with age ≥35 years, and 11.1% with overweight or obesity. The rates of gestational hypertension, preeclampsia and GDM were 3.7, 2.4, and 11.7%, respectively. Rates of C-section and non-medical-indicated C-section were 75.5 and 25.0%, respectively. All newborns had normal 5-min Apgar score (≥8). The mean ± SE of birth weight was 3,430 ± 14 g.

**Table 1:** Characteristics of singleton term newborns (*n* = 922) of mothers with normal thyroid function in a Shanghai birth cohort, China.

**Characteristic**	***N* (%) or Mean ± SE**
Maternal age (years)	29.15 ± 0.11
<30	558 (60.6%)
30–34	311 (33.8%)
≥35	52 (5.6%)
Prepregnancy BMI (kg/m^2^)	21.24 ± 0.10
<18.5	150 (16.3%)
18.5–24.9	668 (72.6%)
≥25	102 (11.1%)
Maternal education	
High school or lower	125 (13.6%)
College/university	796 (86.4%)
Passive smoking during pregnancy	284 (31.1%)
Hypertension	
Chronic hypertension	7 (0.8%)
Gestational hypertension	34 (3.7%)
Preeclampsia	22 (2.4%)
Pre-existed diabetes	3 (0.3%)
GDM	108 (11.7%)
Parity ≥1	90 (9.8%)
Mode of delivery	
Vaginal	226 (24.5%)
C-section (non-medical-indicated)	230 (25.0%)
C-section (medical-indicated)	465 (50.5%)
Infant sex (male)	479 (52.0%)
GA (weeks)	38.95 ± 0.03
Birth weight (g)	3,430 ± 14
LBW	8 (0.9%)
HBW	82 (8.9%)
Birth weight for GA	
SGA	33 (3.6%)
LGA	117 (12.7%)
FT3 (pmol/L)	1.83 ± 0.04
FT4 (pmol/L)	13.37 ± 0.05
FT3/ FT4 ratio	0.16 ± 0.01
TSH (mIU/L)	6.16 ± 0.15
TPOAb ≥5.61 IU/mL	85 (9.3%)

*Data presented are Mean ± SE or n (%)*.

*BMI, body mass index; GDM, gestational diabetes mellitus; C-section, cesarean section; GA, gestational age; LBW, low birth weight; HBW, high birth weight; SGA, small for gestational age; LGA, large for gestational age. FT3, free triiodothyronine; FT4, free thyroxine; TSH, thyroid-stimulating hormone*.

### FT3, FT4, FT3/FT4 Ratio, TSH, and TPOAb Concentrations in Cord Blood

Cord serum FT3 and FT4 concentrations were 0.32 (95%CI: 0.15, 0.49) pmol/L and 0.19 (95%CI: −0.06, 0.44) pmol/L higher, and TSH concentration was 4.27 (95%CI: 3.64, 4.90) mIU/L lower in newborns born via C-section than those born by vaginal deliveries (Table 2). Therefore, cord blood thyroid parameters were further described in C-section and vaginal deliveries separately (Table S1). The mean (SD) cord serum FT3, FT4, and TSH concentrations were 1.90 (1.16) pmol/L, 13.44 (1.66) pmol/L, and 5.15 (2.60) mIU/L in newborns born via C-section, and 1.62 (0.93) pmol/L, 13.17 (1.44) pmol/L and 9.27 (6.76) mIU/L in newborns born via vaginal delivery. Mean (SD) of FT3/FT4 ratio was 0.17 (0.42) in C-section deliveries, and 0.12 (0.07) in vaginal deliveries (Table S1).

**Table 2:** The associations between delivery related factors and cord serum FT3, FT4, and TSH concentrations and FT3/FT4 ratio.

**Variable**	**FT3**		**FT4**		**TSH**		**FT3/ FT4 ratio**	
	**Mean ± SE**	****β** (95%CI)**	**Mean ± SE**	****β** (95%CI)**	**Mean ± SE**	****β** (95%CI)**	**Mean ± SE**	****β** (95%CI)**
Mode of delivery								
Vaginal (*n* = 226)	1.62 ± 0.06	Ref	13.17 ± 0.10	Ref	9.27 ± 0.45	Ref	0.12 ± 0.005	Ref
C-section (*n* = 696)	1.90 ± 0.04	0.32 (0.15, 0.49)^***^	13.44 ± 0.06	0.19 (−0.06, 0.44)	5.15 ± 0.10	−4.27 (−4.90, −3.64)^***^	0.17 ± 0.02	0.05 (−0.01, 0.10)
**Among vaginal deliveries**								
Induction of labor								
No (*n* = 139)	1.70 ± 0.07	Ref	13.14 ± 0.12	Ref	8.06 ± 0.46	Ref	0.13 ± 0.01	Ref
Yes (*n* = 76)	1.42 ± 0.12	−0.28 (−0.56, −0.01)^*^	13.21 ± 0.18	0.03 (−0.40, 0.46)	11.12 ± 0.90	2.85 (0.97, 4.74)^**^	0.11 ± 0.01	−0.02 (−0.04, −0.0003)^*^
Long duration of first stage of labor								
No (*n* = 160)	1.63 ± 0.07	Ref	13.19 ± 0.12	Ref	9.18 ± 0.49	Ref	0.12 ± 0.01	Ref
Yes (*n* = 52)	1.67 ± 0.14	0.03 (−0.26, 0.32)	12.89 ± 0.17	−0.37 (−0.83, 0.09)	10.18 ± 1.22	0.68 (−1.51, 2.88)	0.13 ± 0.01	0.01 (−0.02, 0.03)
Long duration of second stage of labor								
No (*n* = 158)	1.70 ± 0.07	Ref	13.16 ± 0.10	Ref	8.98 ± 0.54	Ref	0.13 ± 0.01	Ref
Yes (*n* = 52)	1.48 ± 0.13	−0.13 (−0.43, 0.17)	13.07 ± 0.26	−0.02 (−0.49, 0.46)	10.82 ± 1.02	2.41 (0.18, 4.65)^*^	0.11 ± 0.01	−0.01 (−0.03, 0.02)

*FT3, free triiodothyronine; FT4, free thyroxine; TSH, thyroid-stimulating hormone*.

*Long duration of first stage of labor: >75th percentile (460 min); Long duration of second stage of labor: >75th percentile (60 min)*.

*All regression models were adjusted for maternal age categories, pre-pregnancy BMI categories, hypertensive disorders of pregnancy, gestational diabetes mellitus or pre-existed diabetes, parity, infant sex and gestational age*.

* p < 0.05;

** p < 0.01;

*** *p < 0.001*.

### Labor-Related Factors and Cord Blood Thyroid Parameters

In vaginal deliveries, cord blood FT3 concentration was 0.28 (95%CI: 0.01, 0.56) pmol/L lower, while TSH concentration was 2.85 (95%CI: 0.97, 4.74) mIU/L higher in newborns born by induction of labor than those by spontaneous labor (Table 2). Cord blood TSH was 2.41 (95%CI: 0.18, 4.65) mIU/L higher in newborns with long duration of second stage of labor (>75th percentile) compared with other infants (Table 2).

### Maternal Age and Cord Blood Thyroid Parameters

Advanced maternal age was associated with lower FT3 in C-section delivery (Table 3). Compared with that in newborns of mothers of age <30 years, cord serum FT3 concentration was 0.15 (95%CI: −0.03, 0.33) pmol/L lower in newborns of mothers aged 30–34 years, and 0.57 (95%CI: 0.22, 0.92) pmol/L lower in newborns of mothers of age ≥35 years in C-section delivery. Similarly, the negative association between maternal age (≥30 years) and cord serum FT3 concentration was observed in vaginal deliveries (*p* = 0.07, Table 3). Cord serum FT3/FT4 ratio was 0.02 (95%CI: 0.0002, 0.04) lower in maternal age ≥30 vs. <30 years in vaginal deliveries, and 0.09 (95%CI: −0.04, 0.22) lower in maternal age ≥35 vs. <30 years in C-section deliveries (Table 3). In sensitivity analysis, negative associations between advanced maternal age and FT3 were observed in non-medical-indicated C-section births (*p* = 0.004, Table S2).

**Table 3:** The associations of maternal age with cord serum concentrations of FT3, FT4, TSH, and FT3/FT4 ratio in C-section and vaginal deliveries.

**Variable**	**FT3**		**FT4**		**TSH**		**FT3/FT4 ratio**	
	**Mean ± SE**	****β** (95%CI)**	**Mean ± SE**	****β** (95%CI)**	**Mean ± SE**	****β** (95%CI)**	**Mean ± SE**	****β** (95%CI)**
**Among C-section deliveries**								
Maternal age (years)								
<30 (*n* = 411)	2.00 ± 0.05	Ref	13.34 ± 0.09	Ref	5.11 ± 0.13	Ref	0.18 ± 0.02	Ref
30–34 (*n* = 238)	1.82 ± 0.08	−0.15 (−0.33, 0.03)	13.59 ± 0.10	0.23 (−0.04, 0.49)	5.23 ± 0.17	0.10 (−0.32, 0.52)	0.16 ± 0.03	−0.02 (−0.08, 0.05)
≥35 (*n* = 46)	1.47 ± 0.15	−0.57 (−0.92, −0.22)^**^	13.63 ± 0.17	0.27 (−0.24, 0.78)	5.06 ± 0.43	−0.003 (−0.82, 0.81)	0.11 ± 0.01	−0.09 (−0.22, 0.04)
P trend	–	**0.002**	–	0.08	–	0.77	–	0.23
**Among vaginal deliveries**								
Maternal age (years)								
<30 (*n* = 147)	1.70 ± 0.07	Ref	13.11 ± 0.11	Ref	9.12 ± 0.54	Ref	0.13 ± 0.01	Ref
≥30 (*n* = 79) ^#^	1.48 ± 0.11	−0.24 (−0.50, 0.02)	13.27 ± 0.17	0.16 (−0.25, 0.56)	9.55 ± 0.81	0.15 (−1.74, 2.04)	0.11 ± 0.01	−0.02 (−0.04, −0.0002)^*^

*FT3, free triiodothyronine; FT4, free thyroxine; TSH, thyroid-stimulating hormone*.

*All models were adjusted for pre-pregnancy BMI categories, hypertensive disorders of pregnancy, gestational diabetes mellitus or pre-existed diabetes, infant sex and gestational age*.

# *Sample size of the group of age ≥35 years in vaginal deliveries was too small (n = 6), so this group was combined into a group of ≥30 years*.

* p < 0.05;

** *p < 0.01*.

Neither advanced maternal age nor mode of delivery was associated with the odds of cord serum TPOAb ≥5.61 IU/mL (Table S3).

### Fetal Growth and Cord Blood Thyroid Parameters

In C-section deliveries, cord blood TSH concentration was 0.87 (95%CI: 0.23, 1.50) mIU/L higher in HBW vs. normal birth weight infants (Table 4). Similar association was observed between LGA and TSH (β = 0.63, 95%CI: 0.08, 1.17). In vaginal deliveries, cord blood TSH concentration was 5.99 (95%CI: 1.21, 10.77) mIU/L higher in SGA vs. AGA infants (Table 4).

**Table 4:** The associations of fetal growth status with cord serum concentrations of FT3, FT4, TSH, and FT3/FT4 ratio in C-section and vaginal deliveries.

**Variable**	**FT3**		**FT4**		**TSH**		**FT3/FT4 ratio**	
	**Mean ± SE**	****β** (95%CI)**	**Mean ± SE**	****β** (95%CI)**	**Mean ± SE**	****β** (95%CI)**	**Mean ± SE**	****β** (95%CI)**
**Among C-section deliveries**								
Birth weight^a^								
LBW (*n* = 7)	1.58 ± 0.43	0.02 (−0.84, 0.88)	13.11 ± 0.58	−0.50 (−1.77, 0.77)	5.23 ± 0.40	0.02 (−1.98, 2.03)	0.12 ± 0.03	0.03 (−0.28, 0.35)
Normal birthweight (*n* = 612)	1.90 ± 0.05	Ref	13.44 ± 0.06	Ref	5.06 ± 0.10	Ref	0.17 ± 0.02	Ref
HBW (*n* = 77)	1.93 ± 0.09	−0.03 (−0.30, 0.24)	13.49 ± 0.24	0.08 (−0.32, 0.48)	5.88 ± 0.44	0.87 (0.23, 1.50)^**^	0.15 ± 0.01	−0.03 (−0.13, 0.07)
Birth weight for GA^b^								
SGA (*n* = 25)	2.02 ± 0.20	0.08 (−0.37, 0.54)	13.13 ± 0.24	−0.28 (−0.95, 0.38)	4.91 ± 0.36	−0.13 (−1.18, 0.92)	0.16 ± 0.01	−0.04 (−0.21, 0.12)
AGA (*n* = 563)	1.89 ± 0.05	Ref	13.43 ± 0.07	Ref	5.06 ± 0.10	Ref	0.17 ± 0.02	Ref
LGA (*n* = 108)	1.95 ± 0.08	0.05 (−0.19, 0.29)	13.59 ± 0.18	0.13 (−0.21, 0.47)	5.70 ± 0.33	0.63 (0.08, 1.17)^*^	0.15 ± 0.01	−0.03 (−0.12, 0.06)
**Among vaginal deliveries**								
Birth weight^a^								
LBW (*n* = 1)		–		–		–		–
Normal birthweight (*n* = 220)	1.64 ± 0.06	Ref	13.17 ± 0.10	Ref	9.09 ± 0.42	Ref	0.12 ± 0.005	Ref
HBW (*n* = 5)	1.30 ± 0.55	−0.28 (−1.11, 0.55)	12.94 ± 0.88	−0.09 (−1.38, 1.20)	9.83 ± 4.63	1.01 (−4.67, 6.70)	0.10 ± 0.04	−0.02 (−0.09, 0.04)
Birth weight for GA^b^								
SGA (*n* = 8)	0.99 ± 0.38	−0.75 (−1.41, −0.09)^*^	12.96 ± 0.51	−0.15 (−1.18, 0.88)	14.66 ± 5.12	5.99 (1.21, 10.77)^*^	0.08 ± 0.03	−0.06 (−0.11, −0.01)^*^
AGA (*n* = 209)	1.66 ± 0.06	Ref	13.15 ± 0.09	Ref	8.98 ± 0.42	Ref	0.13 ± 0.005	Ref
LGA (*n* = 9)	1.34 ± 0.35	−0.28 (−0.90, 0.34)	13.81 ± 1.05	0.68 (−0.28, 1.65)	11.21 ± 3.29	2.30 (−2.19, 6.78)	0.10 ± 0.03	−0.03 (−0.08, 0.02)

*FT3, free triiodothyronine; FT4, free thyroxine; TSH, thyroid-stimulating hormone. LBW, low birth weight; HBW, high birth weight; SGA, small for gestational age; AGA, appropriate for gestational age; LGA, large for gestational age*.

a *Models were adjusted for maternal age categories, pre-pregnancy BMI categories, hypertensive disorders of pregnancy, gestational diabetes mellitus or pre-existed diabetes, infant sex and gestational age*.

b *Models were adjusted for maternal age categories, pre-pregnancy BMI categories, hypertensive disorders of pregnancy, gestational diabetes mellitus or pre-existed diabetes, and infant sex*.

* p < 0.05;

** *p < 0.01; – sample size too small to compute*.

### Prenatal Factors and Cord Blood Thyroid Parameters

There were no significant associations between GDM or hypertensive disorders of pregnancy and cord serum thyroid parameters (Table S4), except that chronic hypertension (*n* = 7) was associated with 2.35 (95%CI: 1.54, 3.16) pmol/L higher cord serum FT3 concentration and 0.92 (95%CI: 0.66, 1.19) higher FT3/FT4 ratio. TPOAb positive was not associated with FT3, FT4, TSH, or FT3/FT4 ratio.

We performed sensitivity analyses to compare the cord blood thyroid parameters in newborns of mothers without any thyroid diseases (*n* = 922) with those with hyperthyroidism (*n* = 10) or hypothyroidism (*n* = 26), and observed no significant differences in cord blood serum FT3, FT4 and TSH levels among the three groups, but the rate of cord blood TPOAb positive (≥ 5.61 IU/mL) was higher in hyperthyroidism (70.0%) and hypothyroidism (46.2%) vs. the normal (9.3%) groups (Table S5). In this population, women with thyroid disorders receive treatment immediately after diagnosis.

## Discussion

We have presented the data on cord serum levels of FT3, FT4, FT3/FT4 ratio, TSH, and TPOAb in a contemporary Chinese term birth cohort. Our study is the first to show that advanced maternal age is associated with lower FT3 in cord blood.

A novel finding of our study was that advanced maternal age was associated with lower FT3 in cord blood. Neonatal thyroid hormone levels are sensitive to variations of maternal thyroid function and iodine status (39, 40). A study also showed that pregnant women of older age had lower FT3 and FT4 levels in the first trimester, and lower levels of FT4 in the second trimester (27). Advanced maternal age has been associated with higher risks of subclinical hypothyroidism (41) and neonatal CH (42) in some studies, but lack of association has also been reported in other studies (43, 44). While it is unclear why advanced maternal age may affect cord blood FT3 levels, we speculate that lower activity of deiodinase type 2 (D2) may be a contributing factor (2, 38). D2 is critical in transforming T4 to FT3 (2). FT3/FT4 ratio is an indicator of D2 activity in converting FT4 into FT3 (38). In addition, type 3 deiodinase (D3) may play a critical role in the decrease in FT3. D3 is upregulated in hypoxia, which is a potential consequence of labor-related adverse events (45). Advanced maternal age has been associated with an increased risk of intrapartum anoxia (46). Neonatal T4 and TSH levels may be associated with cognitive and verbal abilities in children (4, 9, 47–49), and such associations were either positive or negative in previous studies (4, 9, 47–49). A study reported that maternal age > 30 years was associated with lower IQ in the offspring (50). Importantly, our finding suggests that decreased neonatal thyroid hormones may be a potential mechanism in the adverse impact of advanced maternal age on neurodevelopment in the literature. We observed similar associations between maternal age and FT3 in cord blood in C-section and vaginal deliveries. In this study, 52 mothers were 35 years or older, and the sample size was relatively small. However, we did detect a significant effect of advanced maternal age (≥35 years) on cord blood FT3 level.

Gestational age was positively associated with FT3 in cord blood in term infants in our study cohort. In human fetal blood samples (by cordocentesis or percutaneous umbilical cord blood sampling), fetal serum concentrations of FT3 increased from mid-gestation onwards, with an exponential rise closer to term (51), consisting with the progressive increases of fetal FT4 concentrations during pregnancy (2), as the fetal thyroid axis becomes mature during late gestation (1).

We found that high birth weight and LGA were associated with higher TSH. Previous studies have reported positive association (21), or no associations between birth weight and neonatal TSH levels (23, 24). The different findings may be partly due to the differences in ethnicity (8) and measurement method.

Our study confirmed the elevated cord blood TSH levels in newborns of vaginal vs. C-section deliveries (18), and with induction of labor or long duration of second stage of labor (7, 17, 52). Our study also found that newborns born via C-section delivery had higher cord serum FT3 than those born via vaginal delivery. This might be due to the decreased levels of thyroid hormone distributer proteins (THDPs) which include albumin, transthyretin (TTR), and thyroxine-binding globulin (TBG) (53). As albumin, TTR and TBG are negative acute phase plasma proteins, all of them will be down-regulated in situations of stress or surgery (53), resulting in increased FT3. In addition, this can be, in part explained by the role of D3. Since D3 is upregulated in hypoxia, D3 level may rise during vaginal delivery (54), resulting in decrease in T3 and reduction in oxygen consumption (45). A cohort study reported that compared to spontaneous delivery, C-section delivery was associated with lower cord blood serum TSH and FT4 in newborns, but the association disappeared in childhood (around age 6 years) (23).

Cord blood TSH was positively correlated to heel prick blood TSH level (55), which is commonly used in neonatal thyroid function screening (10). The heel prick blood TSH level can be affected by the timing of sample collection and temperature (6, 12). Because newborns commonly experience a physiological surge in TSH levels starting around 30 min after birth until 72 h (56), TSH in heel-prick samples collected between half to 72 h after birth can be higher than cord blood TSH level.

In addition, thyroid hormone levels vary by assay methods with increasing automation, sensitivity and specificity from radioimmunoassay (RIA) to chemiluminescent immunoassay (CLIA) during the last two decades (57, 58). In comparison with other studies using CLIA method in China and in other countries, cord blood serum TSH levels in our study (medians 4.59 mIU/L in C-section delivery, and 7.27 mIU/L in vaginal delivery) was comparable to those reported in Tianjin (median 4.71 mIU/L, *n* = 174) (59), Taiwan (median 4.99 mIU/L, *n* = 107) (60), Shandong (median 8.84 mIU/L, *n* = 107) (61) and Beijing (median 9.44 mIU/L, *n* = 157) (62), as well as the values reported in Belgium (6.6 mIU/L, *n* = 198, Direct CLIA) (63), but lower than those reported in Netherlands (9.42 mIU/L, *n* = 2724, CLIA) (21) and Spain (mean 12.2 mIU/L, *n* = 161, CLIA) (40). Similarly, median cord blood FT3 was around 2 pmol/L and median cord blood FT4 level was 13–15 pmol/L in previous studies and this study (62–66).

We observed no significant association between the presence of TPOAb and FT3, FT4, TSH, or any observed perinatal factors. Neonatal TPOAb was transferred from the mother (67), and is considered normal when undetectable. Some studies linked the presence of TPOAb in cord blood to a higher risk for developing autoimmune thyroiditis in children and adolescents (5).

The study population had a high C-section rate with a large proportion of on-request or non-medical-indicated C-sections. This offers a unique opportunity to assess the cord serum levels of thyroid hormone in physiological conditions. In addition, the relatively large sample size provides robust estimates of cord serum FT3, FT4, TSH, and TPOAb levels in a contemporary Chinese birth cohort. In China, salt iodine fortification is common. The proportion of urinary iodine concentration <150 mg/g creatinine was 34–48% during pregnancy in a Shanghai study population (68). In this study, we excluded women with thyroid diseases before or during pregnancy.

Our study had limitations. This is an observational study, and cannot be used to establish causality. Of note, the assays of FT4 and FT3 can be interfered by thyroid binding protein levels.

## Conclusions

We have presented the data on cord serum levels of thyroid hormones in a Chinese fullterm birth cohort. Our study showed for the first time that advanced maternal age was associated with lower cord blood FT3. Future research is needed to understand whether this association may mediate the association of advanced maternal age with impaired neurodevelopment in early life.

## Data Availability Statement

The datasets generated for this study are available on request to the corresponding author.

## Ethics Statement

The study was reviewed and approved by Medical Ethics Committee of Xinhua Hospital, Shanghai Jiao Tong University School of Medicine. All participants provided their written informed consent to participate in this study.

## Author Contributions

FO conceived and designed the study. FO, JZ, and ZL coordinated and conducted the data collection and measurements. FO and PF analyzed and interpreted data and drafted the manuscript. Z-CL, NT, WW, ZL, and JZ interpreted data and intensively revised the manuscript. All authors have approved the final version of the manuscript.

### Conflict of Interest

The authors declare that the research was conducted in the absence of any commercial or financial relationships that could be construed as a potential conflict of interest.
