# Supplementary material for: Advanced Maternal Age, Mode of Delivery, and Thyroid Hormone Levels in Chinese Newborns
Source: Front Endocrinol (Lausanne). 2020 Jan 10;10:913. doi: 10.3389/fendo.2019.00913 (PMC6966407; doi:10.3389/fendo.2019.00913)
Supplement: Supplementary file 1 [file Table_2.docx]

**Supplemental table S1**. Cord serum FT3, FT4, and TSH concentrations and FT3/FT4 ratio by mode of delivery in Chinese full-term newborns of mothers with normal thyroid function

| **Thyroid parameter** | **Mean (SD)** |  | **Percentiles** | | | | | | | **Range** |
| --- | --- | --- | --- | --- | --- | --- | --- | --- | --- | --- |
|  |  | **2.5th** | | **5th** | **25th** | **50th** | **75th** | **95th** | **97.5th** |  |
| **Among C-section deliveries (n=696)** | |  | |  |  |  |  |  |  |  |
| FT3 (pmol/L) | 1.90 (1.16) | <1.54 | | <1.54 | 1.75 | 2.07 | 2.38 | 2.81 | 2.95 | <1.54 ^a^ – 15.19 |
| FT4 (pmol/L) | 13.44 (1.66) | 10.95 | | 11.31 | 12.67 | 13.41 | 14.29 | 15.93 | 16.41 | <5.15 ^b^ – 18.44 |
| FT3/ FT4 ratio | 0.17 (0.42) | 0 | | 0 | 0.13 | 0.15 | 0.18 | 0.21 | 0.23 | 0 – 6.55 |
| TSH (mIU/L) | 5.15 (2.60) | 2.32 | | 2.59 | 3.69 | 4.59 | 5.73 | 9.13 | 11.85 | 1.73 – 28.87 |
| **Among vaginal deliveries (n=226)** | |  | |  |  |  |  |  |  |  |
| FT3 (pmol/L) | 1.62 (0.93) | <1.54 | | <1.54 | 1.57 | 1.89 | 2.21 | 2.69 | 2.90 | <1.54 ^a^ – 3.73 |
| FT4 (pmol/L) | 13.17 (1.44) | 10.56 | | 11.08 | 12.36 | 13.10 | 14.06 | 15.31 | 16.01 | 7.97 – 21.25 |
| FT3/ FT4 ratio | 0.12 (0.07) | 0 | | 0 | 0.11 | 0.14 | 0.17 | 0.21 | 0.23 | 0 – 0.34 |
| TSH (mIU/L) | 9.27 (6.76) | 2.64 | | 3.13 | 4.96 | 7.27 | 11.07 | 23.17 | 27.32 | 1.91 – 44.93 |

FT3, free triiodothyronine; FT4, free thyroxine; TSH, thyroid-stimulating hormone; SD: standard deviation.

^a^ the limit of detection (LOD) of FT3 is 1.54 pmol/L; ^b^ the LOD of FT4 is 5.15 pmol/L.

**Supplemental table S2.** The associations of maternal age with cord serum concentrations of FT3, FT4, and TSH and FT3/ FT4 ratio in non-medical-indicated C-section deliveries

| **Variable** | FT3 | | FT4 | | TSH | | FT3/ FT4 ratio | |
| --- | --- | --- | --- | --- | --- | --- | --- | --- |
|  | Mean$\pm$SE | $\beta$ (95%CI) | Mean$\pm$SE | $\beta$ (95%CI) | Mean$\pm$SE | $\beta$ (95%CI) | Mean$\pm$SE | $\beta$(95%CI) |
| C-section deliveries ^a^ |  |  |  |  |  |  |  |  |
| Non-medical-indicated (n=230) | 1.89$\pm$0.05 | Ref | 13.53$\pm$0.08 | Ref | 4.98$\pm$0.16 | Ref | 0.14$\pm$0.004 | Ref |
| Medical-indicated (n=465) | 1.91$\pm$0.06 | 0.04 (-0.15, 0.23) | 13.40$\pm$0.09 | -0.15 (-0.43, 0.13) | 5.23$\pm$0.13 | 0.28 (-0.17, 0.72) | 0.18$\pm$0.02 | 0.03 (-0.04, 0.10) |
| **Among non-medical-indicated C-section deliveries** | | |  |  |  |  |  |  |
| Maternal age (years) ^b^ |  |  |  |  |  |  |  |  |
| <30 (n=157) | 1.95$\pm$0.06 | Ref | 13.55$\pm$0.10 | Ref | 5.02$\pm$0.20 | Ref | 0.15$\pm$0.005 | Ref |
| 30-34 (n=66) | 1.83$\pm$0.11 | -0.12 (-0.35, 0.11) | 13.43$\pm$0.16 | -0.10 (-0.46, 0.26) | 4.94$\pm$0.24 | -0.12 (-0.81, 0.57) | 0.14$\pm$0.01 | -0.01 (-0.02, 0.01) |
| $\geq$35 (n=7) | 1.15$\pm$0.42 | -0.89 (-1.50, -0.29)** | 13.94$\pm$0.22 | 0.33 (-0.62, 1.28) | 4.38$\pm$0.65 | -0.58 (-2.41, 1.24) | 0.08$\pm$0.03 | -0.07 (-0.11, -0.02)** |
| P trend | -- | **0.02** | -- | 0.96 | -- | 0.55 | -- | **0.03** |

FT3, free triiodothyronine; FT4, free thyroxine; TSH, thyroid-stimulating hormone; SE: standard error.

^a^ the models were adjusted for maternal age categories, pre-pregnancy BMI categories, hypertensive disorders of pregnancy, gestational diabetes mellitus or pre-existed diabetes, parity, infant sex and gestational age.

^b^ the models were adjusted for pre-pregnancy BMI categories, hypertensive disorders of pregnancy, gestational diabetes mellitus or pre-existed diabetes, infant sex and gestational age.

** p<0.01

**Supplemental table S3.** The associations of mode of delivery and maternal age with cord serum TPOAb

| **Variable** | TPOAb $\geq$5.61 IU/mL | |
| --- | --- | --- |
|  | n (%) | OR (95%CI) |
| Mode of delivery |  |  |
| Vaginal (n=225) | 24 (10.7) | 1.00 (ref) ^a^ |
| C-section (n=690) | 61 (8.8) | 0.84 (0.50, 1.40) |
| **Among C-section deliveries** |  |  |
| Maternal age (years) |  |  |
| <30 (n=407) | 33 (8.1) | 1.00 (ref) ^b^ |
| 30-34 (n=237) | 25 (10.6) | 1.37 (0.79, 2.38) |
| $\geq$35 (n=45) | 3 (6.7) | 0.84 (0.24, 2.97) |
| **Among vaginal deliveries** |  |  |
| Maternal age (years) |  |  |
| <30 (n=147) | 14 (9.5) | 1.00 (ref) ^b^ |
| $\geq$30 (n=78) ^#^ | 10 (12.8) | 1.42 (0.58, 3.53) |

^a^ the model was adjusted for maternal age categories, pre-pregnancy BMI categories, hypertensive disorders of pregnancy, gestational diabetes mellitus or pre-existed diabetes, parity, infant sex and gestational age.

^b^ the model was adjusted for pre-pregnancy BMI categories, hypertensive disorders of pregnancy, gestational diabetes mellitus or pre-existed diabetes, infant sex and gestational age.

^#^ sample size of the group of age $\geq$35 years in vaginal deliveries was too small (n=6), so this group was combined into a group of $\geq$30 years.

**Supplemental table S4.** The associations of hypertensive disorders of pregnancy and diabetes with cord serum concentrations of FT3, FT4, TSH and FT3/ FT4 ratio

| **Variable** | FT3 | | FT4 | | TSH | | FT3/ FT4 ratio | |
| --- | --- | --- | --- | --- | --- | --- | --- | --- |
|  | Mean$\pm$SE | $\beta$ (95%CI) | Mean$\pm$SE | $\beta$ (95%CI) | Mean$\pm$SE | $\beta$ (95%CI) | Mean$\pm$SE | $\beta$(95%CI) |
| Hypertensive disorders of pregnancy | |  |  |  |  |  |  |  |
| No (n=858) | 1.81$\pm$0.04 | Ref | 13.36$\pm$0.05 | Ref | 6.14$\pm$0.15 | Ref | 0.15$\pm$0.01 | Ref |
| Chronic hypertension (n=7) | 4.22$\pm$1.83 | 2.35 (1.54, 3.16)*** | 12.53$\pm$1.73 | -0.90 (-2.10, 0.30) | 5.58$\pm$0.46 | 0.52 (-2.50, 3.54) | 1.08$\pm$0.91 | 0.92 (0.66, 1.19)*** |
| Gestational hypertension (n=34) | 1.85$\pm$0.15 | 0.07 (-0.31, 0.45) | 13.80$\pm$0.25 | 0.36 (-0.19, 0.92) | 6.07$\pm$0.80 | 0.37 (-1.03, 1.76) | 0.14$\pm$0.01 | -0.01 (-0.14, 0.12) |
| Preeclampsia (n=22) | 1.95$\pm$0.17 | 0.10 (-0.36, 0.56) | 13.42$\pm$0.21 | 0.06 (-0.62, 0.74) | 7.18$\pm$1.63 | 1.36 (-0.35, 3.08) | 0.15$\pm$0.01 | -0.01 (-0.16, 0.14) |
| Diabetes mellitus |  |  |  |  |  |  |  |  |
| No (n=810) | 1.83$\pm$0.04 | Ref | 13.38$\pm$0.06 | Ref | 6.18$\pm$0.16 | Ref | 0.15$\pm$0.01 | Ref |
| Pre-existed diabetes (n=3) | 2.24$\pm$0.15 | 0.48 (-0.77, 1.73) | 12.14$\pm$0.92 | -1.44 (-3.27, 0.39) | 9.40$\pm$5.31 | 4.29 (-0.31, 8.89) | 0.18$\pm$0.01 | 0.04 (-0.38, 0.46) |
| GDM (n=108) | 1.85$\pm$0.15 | 0.03 (-0.20, 0.25) | 13.38$\pm$0.19 | -0.06 (-0.38, 0.27) | 5.93$\pm$0.39 | 0.13 (-0.69, 0.95) | 0.19$\pm$0.06 | 0.04 (-0.04, 0.11) |

FT3, free triiodothyronine; FT4, free thyroxine; TSH, thyroid-stimulating hormone; GDM, gestational diabetes mellitus; SE: standard error.

All models were adjusted for mode of delivery (C-section, vaginal delivery) and maternal age categories.

*** p< 0.001

**Supplemental table S5**. Serum concentrations of FT3, FT4, TSH, FT3/ FT4 ratio and positive TPOAb in cord blood of newborns by maternal thyroid function

| **Thyroid parameters** | **Normal**  **(**n=922**)** | **Hypothyroid**  **(**n=26**)** | | **Hyperthyroid**  **(**n=10**)** | | |  |
| --- | --- | --- | --- | --- | --- | --- | --- |
|  | Mean$\pm$SE | Mean$\pm$SE | *p*-Value ^a^ | | Mean$\pm$SE | *p*-Value ^a^ | |
| FT3 (pmol/L) | 1.83$\pm$0.04 | 1.71$\pm$0.16 | 0.57 | | 1.66$\pm$0.39 | 0.62 | |
| FT4 (pmol/L) | 13.37$\pm$0.05 | 13.47$\pm$0.24 | 0.77 | | 12.84$\pm$0.35 | 0.30 | |
| FT3/ FT4 ratio | 0.16$\pm$0.01 | 0.13$\pm$0.01 | 0.09 | | 0.12$\pm$0.03 | 0.31 | |
| TSH (mIU/L) | 6.16$\pm$0.15 | 6.61$\pm$0.94 | 0.61 | | 6.24$\pm$1.05 | 0.96 | |
| TPOAb $\geq$5.61 IU/mL, n (%) | 85 (9.3%) | 12 (46.2%) | **<0.0001** | | 7 (70.0%) | **<0.0001** | |

FT3, free triiodothyronine; FT4, free thyroxine; TSH, thyroid-stimulating hormone; SE: standard error.

^a^ compared with newborns from mother with normal thyroid function.
